# Potential factors affecting semen quality in the Asian elephant (Elephas maximus)

**DOI:** 10.1186/1477-7827-6-9

**Published:** 2008-03-17

**Authors:** Nikorn Thongtip, Jumnian Saikhun, Sittidet Mahasawangkul, Kornchai Kornkaewrat, Pornsawan Pongsopavijitr, Nucharin Songsasen, Anuchai Pinyopummin

**Affiliations:** 1 Faculty of Veterinary Medicine, Kasetsart University, Nakhonpathom 73140, Thailand; 2 Center for Agricultural Biotechnology, Kasetsart University, Nakhonpathom 73140, Thailand; 3 Institute of Science and Technology for Research and Development, Mahidol University, Salaya, Nakhonpathom 73170, Thailand; 4 The National Elephant Institute, Forest Industry Organization, Lampang 52000, Thailand; 5 Faculty of Veterinary Medicine, Chiangmai University, Chiangmai 50200, Thailand; 6 Department of Reproductive Sciences, Conservation and Research Center Smithsonian's National Zoological Park, USA

## Abstract

**Background:**

One of the major obstacles in using artificial insemination to manage genetics of elephant population in captivity is the large variations in semen quality among ejaculates within the same and among individuals. The objectives of this study were to determine the influences of (1) age (2) seasonality (3) and circulating testosterone (SrTest), triiodothyronine (SrT3) and tetraiodothyronine (SrT4), as well as seminal (4) testosterone (SpTest), zinc (SpZn) and protein (SpTP) on semen quality in the Asian elephant

**Methods:**

Analyses, including motility, viability and morphology were performed in semen samples collected twice monthly from 13 elephant bulls (age range, 10-to 72-years) by manual stimulation between July 2004 and June 2005. Serum samples obtained monthly were assessed for SrTest, SrT3, SrT4, and seminal plasma samples were evaluated for, SpTest, SpZn and SpTP.

**Results:**

The highest semen quality was observed at age 23 to 43 years. Percentages of progressive motility and viable sperm were lowest at age 51 to 70 years (P < 0.05); on the other hand, sperm concentration was lowest at age 10 to 19 years (P < 0.05). Percentage of sperm with normal morphology was highest at age 23 to 43 years. The levels of SrT3, SrTest, SpTest and SpZn were lowest at age 51 to 70 years, whereas SrT4 was lowest at age 23 to 43 years. Seasonality significantly affected semen characteristics in which percentage of viable sperm and cell concentration were highest during rainy season and lowest during summer months (P < 0.05). However, percentage of sperm with normal morphology was highest in summer and lowest in rainy season (P < 0.05). Seasonality significantly influenced SrTest with elevated concentrations observed in rainy season and winter (P < 0.05).

**Conclusion:**

This study indicates that age and seasonality had influence on semen characteristics in the Asian elephant. The knowledge obtained in this study will improve our understanding of the reproductive biology of this species.

## Background

Due to the high risks of extinction and increasing concerns regarding the decline of genetic diversity in the ex situ population, efforts have been devoted to the establishment of a self-sustaining population of Asian elephants (*Elephas maximus*) in Thailand. Although the traditional breeding program has been successfully conducted, the ability to perform artificial insemination (AI) in this magnificent species will reduce the risk and cost associated with transporting bulls for natural breeding. The first successful production of live calves after AI with fresh semen in the Asian elephant was demonstrated by Schmitt et al. [1]. Thus far, there is no report on the birth of elephant calves after AI with frozen-thawed spermatozoa, although acceptable post-thaw survival has been reported [2,3]. One of the major obstacles in developing an effective method to cryopreserve Asian elephant spermatozoa is the variation in semen quality of ejaculates obtained from the same or different individuals. A majority of semen samples obtained by manual stimulation exhibit poor quality (i.e., low motility) [2,4], of which the cause has not been determined.

Several studies have suggested that an increase in age is associated with a decline in semen parameters [5-11]. Aging of rodents appears to cause histological changes in the testes, which in turn results in the decline in sperm quality [5,6]. In men, semen volume, sperm concentration, total sperm count, motility, progressive motility, and normal morphology decreased as age increased [7-11]. Moreover, quantitative analysis of sperm motility characteristics using computer assisted sperm analysis (CASA) indicated that age-associated declines in linearity (LIN), straight line velocity (VSL), and average path velocity (VAP) [12].

Seasonality has been shown to affect semen quality in various species, including rams [13], bulls [14], boars [15], bucks [16] and stallions [17]. Seasonal variations in thyroid activity and seminal characteristics have been observed in Iranian fat-tailed rams [18]. Specifically, it was shown that the highest values for thyroid stimulating hormone (TSH), T4, free T4 index, testosterone, total sperm number, percentage of normal sperm, percentage of live sperm, sperm concentration, semen volume and scrotal circumference were observed from early summer to winter and the lowest values were detected at the end of spring [18]. It has been suggested that the thyroid gland may be involved in seasonal transition of reproductive activity in the ram [19]. Low semen quality with decreased sperm concentration and motility and increased percentage of abnormal spermatozoa has also been found in thyroidectomized ram [20]. Based on these observations, we hypothesize that elephant semen quality is associated with the circulating and seminal concentrations of thyroid hormone, in particular T4 and triiodothyronine (T3).

Seminal plasma Zn has been used as a marker for prostatic functions in human [21]. The amount of Zn in the seminal plasma has been shown to be correlated with sperm concentration [22,23], motility and viability [23] in human semen. Seasonal variations in total protein in the seminal plasma were found in boar [15], ram [24] and stallion [25], although species-specific patterns in the profile of total protein content were observed. Total protein in boar ejaculates is highest in fall and winter compared to other seasons. However, total protein in ram seminal plasma is higher in autumn than in summer and winter. Seminal proteins in stallions showed significant differences between pre and post breeding season. Specifically, total protein increases more than 2 fold during the post-breeding season.

Although a number of studies have been conducted to determine factors influencing seminal characteristics in many mammalian species, there are no data available in the Asian elephant. The major goal of this study was to identify possible causes of poor semen quality in the Asian elephants. The specific objectives were to determine the influences of (1) age (2) seasonality (3) circulating reproductive (i.e., SrTest) and thyroid hormones (i.e, SrT3 and SrT4) and (4) SpTest, SpTP and SpZn on seminal parameters.

## Methods

### Chemicals

All chemicals in this study were purchased from Sigma Chemical Company (Sigma, St. Louis, MO, USA) unless stated otherwise.

### Animal and semen collections

All experiments were carried out in strict accordance with the Ethics Committee of the National Elephant Institute. Thirteen Asian elephant bulls (EM1 to EM13) (age range from 10-to 72-years-old) housed at the National Elephant Institute, Forest Industry Organization, Lampang, Thailand were included in this study. The elephants were fed with grass, banana and sugar cane during the day and allowed to roam in the jungle at night. Semen samples from each bull were collected twice monthly by manual manipulation as described by Schmitt and Hildebrandt [26] starting from July 2004 through June 2005 except in September when semen collection was not performed. During the course of this study, 4 bulls were in musth: EM1 (November 2004), EM5 (November 2004), EM11 (January 2005) and EM12 (December 2004 to January 2005). Musth was characterized by aggressive behaviour and the presence of secretions from temporal gland and prepuce. Due to safety concerns, semen and serum samples were not collected from these bulls during musth periods. Each ejaculate was immediately evaluated for volume, sperm concentration, progressive motility, sperm viability and pH [27]. Sperm concentration was assessed using a haemocytometer. Progressive motility was assessed visually under a phase-contrast microscopy by two independent technicians. Sperm viability was assessed using an eosin-nigrosin staining method (200 spermatozoa were counted per slide at 1000× magnification and classified as dead [stained] or live [unstained] [28].

### Hormonal assay

#### Serum triiodothyronine (T3) and tetraiodothyronine (T4)

Serum samples were collected monthly from all bulls (i.e. 11 samples of non musth bulls; 10 samples of EM1, EM5, EM11 and 9 samples from EM12). In each case, 10 ml blood was collected from an ear vein using a vacuum tube (Venoject^R^, Terumo, Tokyo, Japan) and allowed to clot at room temperature for 1 to 2 h before centrifugation at 1,000 g for 10 min. The serum was aspirated and then frozen in 1.5 ml aliquots and stored at -20°C until analysis. Serum T3 and SrT4 were analyzed using automated chemiluminescence system (ACS: 180, Bayer Corporation, Tarrytown, NY, USA).

#### Seminal plasma and serum testosterone

Seminal plasma was collected monthly from all bulls by centrifugation of the semen samples at 1,000 g for 10 min. The supernatant fluids were collected and stored at -20°C until analysis. Before analysis, seminal plasma was diluted (1:50) by assay buffer [0.04 M sodium phosphate (monobasic, monohydrate), 0.06 M sodium phosphate (dibasic), 0.87% NaCl and 0.1% BSA, pH 7.0]. Anti-testosterone was diluted (1:100) in coating buffer (0.035 M sodium bicarbonate and 0.015 M sodium carbonate, pH 9.6). Seminal plasma and serum testosterone were assessed using an enzyme immunoassay previously validated for elephant serum [29]. Polyclonal anti-testosterone R156/7 was kindly provided by Dr. Janine L. Brown, Conservation and Research Center, Smithsonian Institute, USA. A two-dimensional titer determination for optimum dilution of anti-testosterone was 1:10,000 and testosterone HRP was 1:15,000. Precision of the assay was assessed by determination of intra-assay (measurement of the same control samples within one assay) and inter-assay coefficients of variations (CV). The CVs for intra- and inter-assays were 9% and 12%, respectively. Sensitivity was determined by calculating the first standard value (ng/ml) that differed significantly from the absorbance of the zero standard. The testosterone sensitivity was 0.046 ng/ml.

#### Seminal plasma Zn and total protein

Seminal plasma Zn was analyzed using flame atomic absorption spectrophotometry (FAAS) (Varian 220Z, Varian, Australia) with standard solution Zn 1000 ppm (FGS Chemicals, Powell, OH, USA) [30]. Seminal total protein was analyzed by using Biuret reaction [31] via cassette COBAS INTEGRA Total Protein kit on COBAS INTEGRA 700 (Roche Diagnostics, Basel, Switzerland). The sensitivity was 4.8 × 10^-2 ^ΔA per g/dl of total protein.

### Statistical analysis

The data were expressed as mean ± SE, and analyzed using SPSS 13.0 software (SPSS Inc., Chicago, IL, USA). Age was divided into three groups according to their maturity; 10 to 19, 23 to 43 and 51 to 70 years old. Seasonality was separated into three seasons; rainy (July to October 2004), winter (November 2004 to February 2005) and summer (March to June 2005). The comparisons of mean ± SE of seminal and serum parameters among ages and seasons were performed using Mann-Whitney U or Wilcoxon Rank-Sum Test for differences among medians. The comparisons of mean ± SE of seminal and serum parameters obtained from elephants in groups with moderate or low-motile sperm in each season were performed by using t-test. Differences were considered significance when P < 0.05.

## Results

A total of 286 attempts were made to collect semen from 13 elephant bulls between July 2004 and June 2005. Of 286 attempts, 226 (79.0%) were successful. Ejaculates were obtained from all semen donors but 51 (22.6%) out of 226 ejaculates were contaminated with urine and discarded from the study. Overall the 175 ejaculates used in this experiment showed high variations of seminal characteristics. Age of semen donors significantly affected seminal parameters and hormonal profiles (Table 1). Specifically, percentages of progressive motility, viable sperm and SpZn of samples obtained from 51 to 70 years old bulls were significantly lower than in younger groups (P < 0.05). However, sperm concentration was significantly lower in samples obtained from young bulls (10 to 19 years of age) than those obtained from older individuals (P < 0.05). The highest percentage of sperm with normal morphology was observed in samples obtained from bulls at age 23 to 43 years. Aging bulls had significantly lower circulating T3 than young ones; serum T4 of the oldest group was similar to that of youngest group, but higher than the value for middle age bulls. Serum testosterone was not affected by age; however, seminal testosterone concentration was higher in young bulls compared to older individuals. Mean values of seminal and serum parameters assessed during the three seasons are summarized in Table 2. Seasonality significantly affected semen characteristics, including sperm concentration, percentage of viable sperm and percentage of sperm with normal morphology but did not affect other parameters. Ejaculates obtained during rainy season and winter contained a higher proportion of viable spermatozoa than those collected during summer period (P < 0.05). Sperm concentration of ejaculates obtained during the rainy season was significantly higher (P < 0.05) than that obtained during summer and winter. In contrast, the percentage of sperm with normal morphology of samples obtained in rainy season was lower (P < 0.05) than those obtained during other seasons. With the exception of SrTest, seasonality has no influence on serum and seminal hormones as well as SpTP and SpZn. SrTest was significantly higher (P < 0.05) in samples obtained during the rainy season and winter than those obtained in summer.

**Table 1:** Seminal and serum parameters of samples obtained during July 2004 through June 2005 in 10 to 19 (n = 3), 23 to 43 (n = 8) and 51 to 70 (n = 2) years old bulls.

	Age group (years)		
	
Parameters	10–19 (27 ejaculates)	23–43 (118 ejaculates)	51–70 (30 ejaculates)
Progressive motility (%)	22.78 ± 3.78^a^	31.28 ± 2.11^a^	0.00 ± 0.00^b^
Concent. (× 10^6 ^/ml)	1089.86 ± 168.99 ^a^	1502.03 ± 91.90^b^	1705.5 ± 199.95 ^b^
Volume (ml)	18.18 ± 3.07	24.00 ± 1.70	20.21 ± 3.50
Seminal pH	7.84 ± 0.19	7.78 ± 0.11	8.27 ± 0.22
Viable sperm (%)	46.50 ± 3.60 ^a^	33.70 ± 3.10 ^a^	27.10 ± 3.50^b^
Normal morphology (%)	73.12 ± 3.71^a^	84.48 ± 2.17^b^	66.98 ± 4.30^a^
SrT3 (ng/dl)	224.86 ± 9.61^a^	169.54 ± 5.68^b^	138.95 ± 11.66^c^
SrT4 (μg/dl)	11.51 ± 0.31^a^	9.81 ± 0.18^b^	10.63 ± 0.38^a^
SrTest (ng/ml)	14.90 ± 6.49	8.6 ± 1.92	2.43 ± 0.67
SpTest (ng/ml)	68.11 ± 7.61^a^	46.89 ± 3.76^b^	40.99 ± 8.20^b^
SpTP (g/dl)	0.48 ± 0.08	0.63 ± 0.04	0.60 ± 0.08
SpZn (μg/dl)	53.29 ± 5.12^a^	61.34 ± 2.37^a^	39.23 ± 5.39^b^

Data were mean ± SE, superscripts within the same parameters differ significantly (P < 0.05)

**Table 2:** Seasonal variations of semen characteristics and hormonal profiles in serum and seminal plasma, SpTP and SpZn collected from 13 elephant bulls during rainy (July to October 2004), winter (November 2004 to February 2005) and summer (March to June 2005).

Parameters	Rainy (50 ejaculates)	Winter (70 ejaculates)	Summer (55 ejaculates)
Progressive motility (%)	25.70 ± 3.40	23.20 ± 3.10	26.50 ± 3.10
Concentration (x10^9^/ml)	1.86 ± 0.14^a^	1.29 ± 0.12^b^	1.26 ± 0.12^b^
Volume (ml)	23.90 ± 2.50	23.20 ± 3.10	19.30 ± 2.30
Semen pH	7.80 ± 0.14	7.80 ± 0.25	7.90 ± 0.13
Viable sperm (%)	46.50 ± 3.60 ^a^	33.70 ± 3.10 ^a^	27.10 ± 3.50^b^
Normal morphology (%)	64.94 ± 2.84^a^	84.53 ± 2.75^b^	86.82 ± 2.52 ^b^
SrT3 (ng/dl)	170.20 ± 9.62	182.90 ± 8.42	176.70 ± 8.70
SrT4 (μg/dl)	10.60 ± 0.29	9.89 ± 0.26	10.47 ± 0.27
SrTest (ng/ml)	20.20 ± 5.69 ^a^	23.70 ± 5.25 ^a^	6.10 ± 5.19^b^
SpTest (ng/ml)	47.50 ± 7.60	54.40 ± 4.80	45.30 ± 5.00
SpTP (g/dl)	0.71 ± 0.07	0.58 ± 0.04	0.57 ± 0.05
SpZn (μg/dl)	52.30 ± 4.80	55.90 ± 3.10	61.10 ± 3.50

Data were mean ± SE, superscripts within the same parameters differ significantly (P < 0.05)

**Figure 1:**
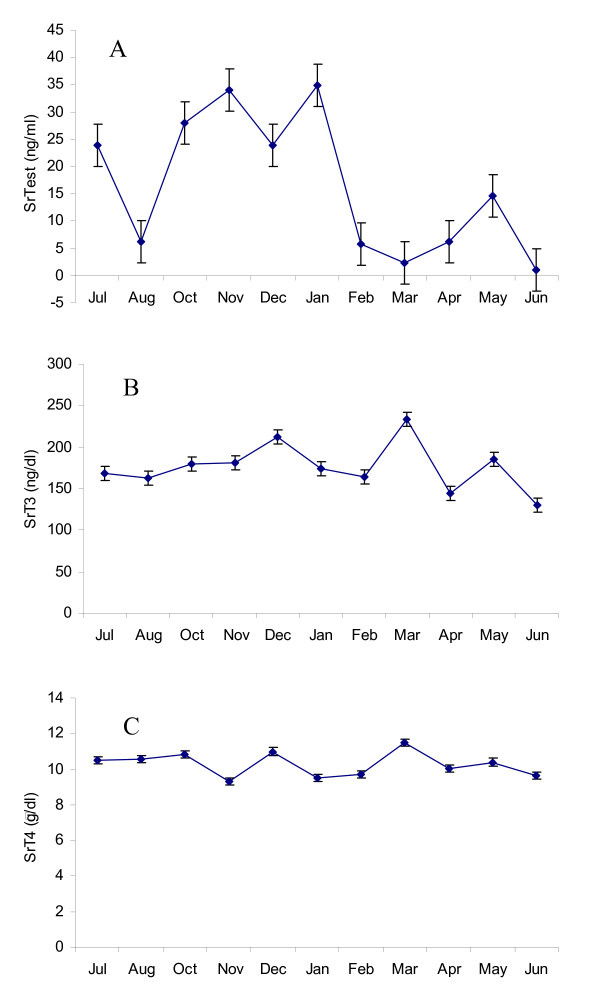
Seasonal variations of serum (A) testosterone (SrTest), (B) triiodothyronine (SrT3) and (C) tetraiodothyronine (SrT4) in the Asian elephant bulls collected between July 2004 and June 2005.

**Figure 2:**
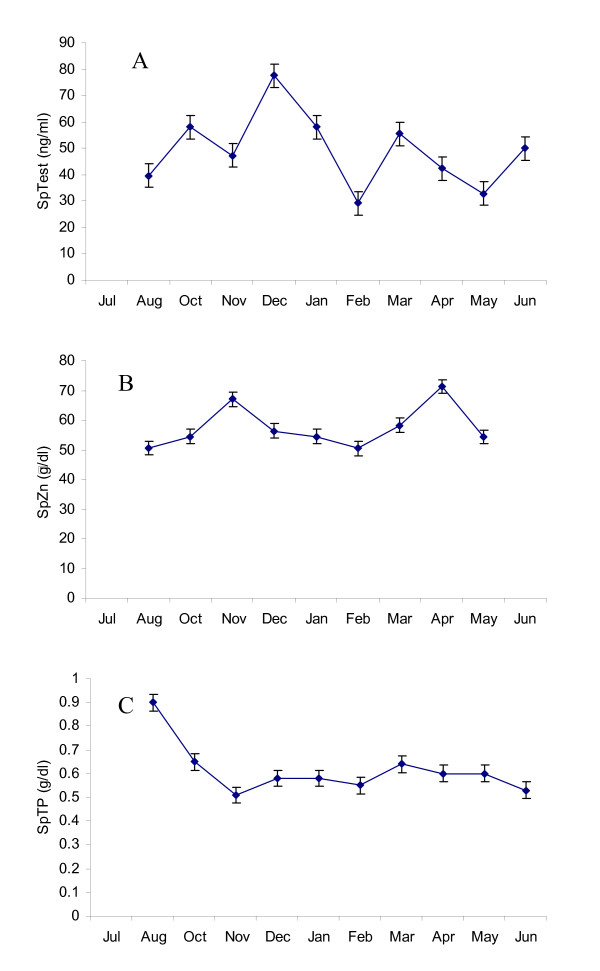
Seasonal variations of seminal plasma (A) testosterone (SpTest), (B) zinc (SpZn) and (C) total protein (SpTP) in the Asian elephant bulls collected between July 2004 and June 2005.

Fig 1 depicts SrTest (A), SrT3 (B) and SrT4 (C) levels assessed between July 2004 and June 2005. Serum testosterone started to increase in October and maintained high levels in the winter months until January and then significantly declined to a lower level in February and remained at this level until June. Circulating T4 and T3, as well as SpTest, SpTP and SpZn did not vary among seasons (Figs 1 and 2). With the exception of EM12, progressive motility of samples collected from bulls exhibiting musth was higher during the month prior to the initiation of musth period except for EM12. Progressive motility of spermatozoa from EM1 and EM5 decreased after the musth period, but was maintained at a high level in the month after musth in EM11.

Due to high variation of semen characteristics obtained from individual bulls, we have divided elephant bulls into 2 groups according to their progressive motility to examine the relationship between semen characteristics and serum and seminal hormones as well as SpTP and SpZn obtained in different seasons. The elephant bulls that yielded spermatozoa with 0 to 10% and 30 to 50% progressive motility were classified as low- and moderate-motile groups, respectively. A direct comparison of semen quality and serum and seminal hormones as well as SpTP and SpZn between low-and moderate-motile groups is shown in Table 3. Overall seminal and hormonal parameters were significantly different between the two groups; however, seasonality influenced these variations. Percentage of viable sperm in moderate-motile group was significantly higher (P < 0.05) than that in low-motile group in winter and summer; no significant difference between groups was found in samples collected during rainy season. Seminal volume in moderate-motile group was also significantly higher (P < 0.05) than that of low-motile group, especially in summer. Percentage of sperm with normal morphology and SrT3 in moderate-motile group were significantly higher (P < 0.05) than that in low-motile group in rainy and winter. The levels of SpTP and SpZn in moderate-motile group were also significantly higher (P < 0.05) than that in low-motile group in winter and summer.

**Table 3:** Semen characteristics and hormonal profiles in serum and seminal plasma, SpTP and SpZn in low-motile and moderate-motile semen collected from 13 elephant bulls during rainy (July to October 2004), winter (November 2004 to February 2005) and summer (March to June 2005).

Semen parameters	Rainy		Winter		Summer	
	
	Moderate-motile (33 ejaculates)	Low-motile (17 ejaculates)	Moderate-motile (46 ejaculates)	Low-motile (24 ejaculates)	Moderate-motile (31 ejaculates)	Low-motile (24 ejaculates)
Progressive motility (%)	39.05 ± 4.27^a^	6.7 ± 3.08^b^	37.14 ± 4.16^a^	5.83 ± 2.65^b^	37.84 ± 4.65^a^	0.00 ± 0.00 ^b^
Concentration (x10^6^/ml)	1940 ± 183.82	1870 ± 385.45	1080 ± 99.66	1280 ± 301.44	1210 ± 145.06	1090 ± 166.08
Volume (ml)	28.95 ± 4.35	17.40 ± 4.56	25.95 ± 3.63	21.11 ± 4.15	23.84 ± 2.76^a^	10.27 ± 1.64^b^
Seminal pH	7.59 ± 0.14	8.25 ± 0.17	7.95 ± 0.25	8.33 ± 0.21	7.85 ± 0.18	8.50 ± 0.16
Viable sperm (%)	48.68 ± 4.51	47.17 ± 31.16	40.66 ± 4.48^a^	26.59 ± 6.56^b^	36.84 ± 3.94^a^	6.36 ± 2.30^b^
Normal morphology (%)	79.60 ± 8.20^a^	55.00 ± 10.10^b^	88.50 ± 1.60^a^	79.50 ± 2.50^b^	88.70 ± 1.70	86.50 ± 3.00
SrT3 (ng/dl)	191.81 ± 12.49^a^	137.87 ± 10.16^b^	197.53 ± 11.48^a^	148.39 ± 13.83^b^	179.46 ± 12.92	154.49 ± 16.22
SrT4 (μg/dl)	10.87 ± 0.44	10.88 ± 0.33	10.03 ± 0.32	10.50 ± 0.55	10.50 ± 0.36	10.95 ± 0.52
SrTest (ng/ml)	14.05 ± 5.19	6.97 ± 1.47	18.41 ± 4.39	8.91 ± 2.09	2.57 ± 0.68	1.03 ± 0.30
SpTest (ng/ml)	47.35 ± 5.04	54.38 ± 10.67	51.15 ± 6.41	65.07 ± 18.00	46.93 ± 4.65	44.68 ± 6.09
SpTP (g/dl)	0.62 ± 0.08	0.77 ± 0.32	0.64 ± 0.05^a^	0.49 ± 0.11^b^	0.57 ± 0.05^a^	0.28 ± 0.06^b^
SpZn (μg/dl)	51.47 ± 4.64	49.82 ± 5.93	56.29 ± 3.50^a^	46.33 ± 5.78^b^	60.69 ± 3.58^a^	50.37 ± 9.18^b^

Data were mean ± SE, superscripts within the same parameters differ significantly (P < 0.05)

## Discussion

Seminal characteristic variations within and among individual bulls are the major obstacle for developing reliable cryopreservation methods in the Asian elephant. We demonstrate in the present study that, elephant age affected seminal characteristics and hormonal profiles in serum and seminal plasma. Our findings are consistent with those in rodent [5,6] and human [7-11] showing that semen quality declined with age. Wang et al. [5] reported that total sperm production was significantly reduced in older rats (22 and 30 months old). Eskenazi et al [10] reported that semen volume and sperm motility in healthy men decreased continuously between 22 and 80 years of age. Normal sperm morphology according to the World Health Organisation (WHO) criteria was significantly lower in patients aged > 45 years [11]. Recently, Levitas et al [32], reported that the best seminal parameters were observed in men from 30 to 35 years old whereas significant reduction in sperm quality occurred after the age of 55. The age-associated decline in semen quality in this study may be due to changes in functions of the testes and/or the endocrine system. Parkening et al. [33] found that older mice had atrophic testes, fewer motile sperm, and degenerated seminiferous epithelium. Tanemura et al. [6] reported that vacuoles appeared in germ cells and cell numbers decreased in older mice (18 months old), resulting in a thinner seminiferous epithelium. Spermatids and spermatocytes essentially disappeared in very old mice (33 months old), as spermatogenesis was severely disrupted. Moreover, age-related histological alterations in the testis include a reduction in the number of Leydig cells [34] which are known to be responsible for testosterone production. It has been reported that free testosterone and albumin-bound testosterone in men decline by 50% between ages 20 and 80 years [35]. Histological alterations of the testes in aging males probably are associated with the decline in blood testosterone, which in turn results in the decreases in other physiologic functions, including bone density, muscle strength and libido [36].

We also found that semen quality varied among seasons. Our finding that sperm concentration and viability were lowest in samples obtained during summer is in agreement with those of previous reports in the human [37] and pig [38]. However, our finding is in contrast to those observed in water and swamp buffalo [39,40] of which optimal seminal quality was observed in samples obtained during rainy season and summer. The species differences between elephants and buffaloes may be due to variations in study location and techniques used for semen analysis. The explanation for lower sperm concentration and viable spermatozoa in summer compared to other seasons may be due to ambient temperature. Spermatogenesis is a highly temperature-sensitive process. Sperm production in humans is known to decrease when testicular temperature is raised by experimental techniques [41]. Thailand is located in the tropical area and temperature can reach to 41.5°C in summer [42]. We speculate that the rise in ambient temperature in summer may affect sperm production [37] and sperm viability as has been reported in mice [43] and wild boars [44]. However, temperature may not influence sperm morphology, since a study on semen quality of swamp buffalo bulls used for artificial insemination under tropical conditions in Thailand showed that the proportion of morphologically normal spermatozoa were highest in summer [40] and a similar finding was observed in Asian elephant bulls in the present study. A complicating factor is that testes in elephants are intra-abdominal unlike the situation in other mammalian species where the testes are located in the scrotum to avoid damage to sperm from high body temperatures. Thus, the high temperature inside and intra-abdominal testes of elephants might affect sperm production and the longevity of elephant spermatozoa. Further studies are required to investigate this hypothesis.

Serum and seminal hormones did not exhibit any seasonal variation except for SrTest concentration which was higher in winter and rainy season than in summer. Variations in SrTest among seasons may be due to food availability and animal health. In the rainy season, the elephants obtained a higher quality diet, while foraging in the jungle than during the summer season. Seasonal fluctuation in body condition was associated with variation in feed resource and positively correlated to folliculogenesis in females [45]. The serum testosterone profiles was associated with musth and appeared to be influenced by nutrition in Asian elephant bulls [46].

The levels of SrTest and SrT3 were associated with elephant semen quality. The levels of circulating testosterone and T3 were higher in the group of individuals with better seminal quality. The correlation between SrT3 and SrTest, as well as their association with semen quality has been previously described in other mammalian species. T3 directly regulates Sertoli and Leydig cell functions which in turn indirectly modulate testosterone synthesis by Leydig cells [47]. It has been shown that the peak of plasma T3 levels coincided with the rise of testosterone concentrations in domestic ganders [48]. In Iranian fat-tailed rams, the peak circulating T3 was observed when testosterone reached the maximum level and corresponding to the time of the year when sexual activity reached its highest level [18]. Serum T3 also exhibited a positive correlation with total sperm concentration and percentage of live spermatozoa [39]. It has been reported that the mean value for testosterone was significantly lower in oligospermic and azoospermic men as compared to normospermic control groups [49]. Recently, Meeker et al. [50] found that there was a suggestive positive association between testosterone and human sperm motility.

It was found that moderate-motile group had higher concentrations of zinc and total protein in the seminal plasma than did those in low-motile group. This finding agreed with those reported in previous studies in other mammalian species, including the human, cattle and pig [49,51,52]. In human, serum and seminal plasma zinc levels were low in oligospermic, and azoospermic subjects when compared with normospermic control groups [49,53]. Similarly, sperm concentration, live sperm and motility were significantly higher in Zn-supplemented bulls as compared to the control group [51]. In boar semen, a high content of seminal total protein was also associated with the increase in percentages of spermatozoa with intact plasma membrane [52]. It has been reported that sperm motility and sperm concentration and testosterone level were correlated with total protein in the seminal plasma [54].

## Conclusion

We have conducted a milestone study that will improve our understanding of the reproductive biology of Asian elephant bulls. We demonstrate that bull age and seasonality significantly influence gonadal, thyroidal activity and seminal quality. Clearly, more knowledge on sperm biology in this species needed to be obtained to allow us to fully understand the cause of large variations in semen quality among ejaculates. Such information is crucial for the development of an effective method to cryopreserve elephant semen for genetic management of this endangered species in captivity.

## Competing interests

The author(s) declare that they have no competing interests.

## Authors' contributions

NT, JS and KK participated in all aspects of the experiment. PP participated in hormonal analysis. NS and AP participated in experiment design and edited the manuscript. All authors read and approved the final manuscript.

## References

[B1] Schmitt DL Hildebrant TB Hermes R Goritz F Assisted reproductive technology in elephants Proc 1st Int Symp Assisted Reproductive Technology for Conservation Genetic Management of Wildlife, Omaha 's Henry Doorly Zoo 2001 15 17

[B2] Thongtip N Saikhun J Damyang M Mahasawangkul S Suthunmapinata P Yindee M Kongsila A Angkawanish T Jansittiwate S Wongkalasin W Wajjwalkul W Kitiyanant Y Pavasuthipaisit K Pinyopummin A Evaluation of post-thaw Asian elephant (*Elephas maximus*) spermatozoa using flow cytometry: the effects of extender and cryoprotectant Theriogenology 2004 62 748 760 10.1016/j.theriogenology.2003.11.021 15226027

[B3] Sa-ardrit M Saikhun J Thongtip N Damyang M Mahasawangkul S Angkawanish T Jansittiwate S Kitiyanant Y Pavasuthipaisit K Pinyopummin A Ultrastructural alterations of frozen-thawed Asian elephant (*Elephas maximus*) spermatozoa Int J Androl 2006 29 346 352 10.1111/j.1365-2605.2005.00578.x 16533357

[B4] Thongtip N Sanyathitiseree P Damyang M Theerapan W Suthummapinunta P Mahasawangkul S Angkawanish T Jansittiwate S Pinyopummin A The preliminary study of semen evaluation from Thai captive elephants39th Kasetsart University Animal Conference, Bangkok 2001 312 315

[B5] Wang C Leung A Sinha-Hikim AP Reproductive aging in the male brown-Norway rat: a model for the human Endocrinology 1993 133 2773 2781 10.1210/en.133.6.2773 8243304

[B6] Tanemura K Kurohmaru M Kuramoto K Hayashi Y Age-related morphological changes in the testis of the BDF1 mouse J Vet Med Sci 1993 55 703 710 8286519 10.1292/jvms.55.703

[B7] Centola GM Eberly S Seasonal variations and age-related changes in human sperm count, motility, motion parameters, morphology, and white blood cell concentration Fertil Steril 1999 72 803 808 10.1016/S0015-0282(99)00395-7 10560981

[B8] Kidd SA Eskenazi B Wyrobek AJ Effects of male age on semen quality and fertility: a review of the literature Fertil Steril 2001 75 237 248 10.1016/S0015-0282(00)01679-4 11172821

[B9] Chen Z Toth T Godfrey-Bailey L Mercedat N Schiff I Hauser R Seasonal variation and age-related changes in human semen parameters J Androl 2003 24 226 231 12634309 10.1002/j.1939-4640.2003.tb02666.x

[B10] Eskenazi B Wyrobek AJ Sloter E Kidd SA Moore L Young S Moore D The association of age and semen quality in healthy men Hum Reprod 2003 18 447 454 10.1093/humrep/deg107 12571189

[B11] Pasqualotto FF Sobreiro BP Hallak J Pasqualotto EB Lucon AM Sperm concentration and normal sperm morphology decrease and follicle-stimulating hormone level increases with age BJU Int 2005 96 1087 1091 10.1111/j.1464-410X.2005.05806.x 16225533

[B12] Sloter E Schmid TE Marchetti F Eskenazi B Nath J Wyrobek AJ Quantitative effects of male age on sperm motion Hum Reprod 2006 21 2868 2875 10.1093/humrep/del250 16793993

[B13] Ibrahim SA Seasonal variations in semen quality of local and crossbred rams raised in the United Arab Emirates Anim Reprod Sci 1997 49 161 167 10.1016/S0378-4320(97)00063-8 9505109

[B14] Parkinson TJ Seasonal variation in semen quality of bulls and correlations with metabolic and endocrine parameters Vet Rec 1985 117 303 307 4060537 10.1136/vr.117.12.303

[B15] Trudeau V Sanford LM Effect of season and social environment on testis size and semen quality of the adult Landrace boar J Anim Sci 1986 63 1211 1219 3771402 10.2527/jas1986.6341211x

[B16] Asher GW Berg DK Evans G Storage of semen and artificial insemination in deer Anim Reprod Sci 2000 62 195 211 10.1016/S0378-4320(00)00159-7 10924825

[B17] Roser JF Hughes JP Seasonal effects on seminal quality, plasma hormone concentrations, and GnRH-induced LH response in fertile and subfertile stallions J Androl 1992 13 214 223 1318293

[B18] Zamiri MJ Khodaei HR Seasonal thyroidal activity and reproductive characteristics of Iranian fat-tailed rams Anim Reprod Sci 2005 88 245 255 10.1016/j.anireprosci.2004.12.005 16143215

[B19] Parkinson TJ Follett BK Effect of thyroidectomy upon seasonality in rams J Reprod Fert 1994 101 1 51 58 10.1530/jrf.0.1010051 8064693

[B20] Brookes JR Ross CF Turner CW Effect of thyroidectomy on reproductive performance of ewes and semen quality of rams J Anim Sci 1965 25 55 58

[B21] Ahlgren G Rannevik G Lilja H Impaired secretory function of the prostate in men with oligo-asthenozoospermia J Androl 1995 16 491 498 8867597

[B22] Xu B Chia SE Tsakok M Ong CN Trace elements in blood and seminal plasma and their relationship to sperm quality Reprod Toxicol 1993 7 613 618 10.1016/0890-6238(93)90038-9 8118112

[B23] Chia SE Ong CN Chua LH Ho LM Tay SK Comparison of zinc concentrations in blood and seminal plasma and the various sperm parameters between fertile and infertile men J Androl 2000 21 53 57 10670519

[B24] Gundogan M Some reproductive parameters and seminal plasma constituents in relation to season in Akkaraman and Awassi Rams Turk J Vet Anim Sci 2006 30 95 100

[B25] Kosiniak K Bittmar A Biochemical components of stallion seminal plasma before and after the breeding season Anim Reprod Sci 1981 4 39 47 10.1016/0378-4320(81)90018-X

[B26] Schmitt DL Hildebrandt TB Manual collection and characterization of semen from Asian elephants (*Elephas maximus*) Anim Reprod Sci 1998 53 309 314 10.1016/S0378-4320(98)00120-1 9835384

[B27] Hafez ESE Hafez ESE Semen evaluation Reproduction in Farm Animals 1993 5 Philadelphia: Lea and Febiger 405 423

[B28] BjoÃ rndahl1 L SoÃ derlund I Kvist U Evaluation of the one-step eosin-nigrosin staining technique for human sperm vitality assessment Hum Reprod 2003 18 813 816 10.1093/humrep/deg199 12660276

[B29] Brown JL Walker S Steinman K Endocrine manual for reproductive assessment of domestic and non-domestic species Conservation and Research Center, Smithsonian's National Zoological Park Endocrine Workshop on Reproductive Assessment of Domestic and Non-domestic Species. Faculty of Veterinary Medicine, Chiang Mai University, Thailand 2004 53 55

[B30] Eggert-Kruse W Zwick E Batschulat K Rohr G Armbruster FP Petzoldt D Strowitzki T Are zinc levels in seminal plasma associated with seminal leukocytes and other determinants of semen quality? Fertil Steril 2002 77 260 269 10.1016/S0015-0282(01)02974-0 11821081

[B31] Doumas BT Bayse DD Carter RJ Peters JR Schaffer RA Candidate reference method for determination of total protein in serum. I. Development and validation. II. Tests for transferability Clin Chem 1981 27 1642 1654 6169466

[B32] Levitas E Lunenfeld E Weisz N Friger M Potashnik G Relationship between age and semen parameters in men with normal sperm concentration: analysis of 6022 semen samples Andrologia 2007 39 45 50 10.1111/j.1439-0272.2007.00761.x 17430422

[B33] Parkening TA Collins TJ Au WW Paternal age and its effects on reproduction in C57BL/6NNia mice J Gerontol 1988 43 3 79 84 10.1093/geronj/43.3.b79 3361087

[B34] Neaves WB Jonson L Porter JC Parker CR Jr Petty CS Leydig cell number, daily sperm production and serum gonadotropin levels in aging men J Clin Endocr Metab 1984 59 756 763 6434579 10.1210/jcem-59-4-756

[B35] Herman M Berger P Ageing of male endocrine system Rev Physiol Biochem Pharm 1999 139 90 122 10.1007/BFb0033649 10453693

[B36] Schubert M Jockenhovel F Late-onset hypogonadism in the aging male: definition, diagnostic and clinical aspects J Endocrinol Invest 2005 28 23 27 16042356

[B37] Levine RJ Mathew RM Chenault CB Brown MH Hurtt ME Bentley KS Mohr KL Working PK Differences in the quality of semen in outdoor workers during summer and winter N Engl J Med 1990 323 12 16 2355953 10.1056/NEJM199007053230103

[B38] Ciereszko A Ottobre JS Glogowski J Effects of season and breed on sperm acrosin activity and semen quality of boars Anim Reprod Sci 2000 64 89 96 10.1016/S0378-4320(00)00194-9 11078969

[B39] Dixit NK Agrmal SP Agarwal VK Dwarakna PK Seasonal variation in serum level of thyroid hormones and their relation with seminal quality and libido in buffalo bulls Theriogenology 1984 22 497 507 10.1016/0093-691X(84)90049-9 16725982

[B40] Koonjaenak S Chanatinart V Aiumlamai S Pinyopumimintr T Rodriguez-Martinez H Seasonal variation in semen quality of swamp buffalo bulls (*Bubalus bubalis*) in Thailand Asian J Androl 2007 9 92 101 10.1111/j.1745-7262.2007.00230.x 17187160

[B41] Mieusset R Bujan L Mansat A Pontonnier F Grandjean H Hyperthermia and human spermatogenesis: enhancement of the inhibitory effect obtained by 'artificial cryptorchidism Int J Androl 1987 10 571 580 2888735 10.1111/j.1365-2605.1987.tb00356.x

[B42] Thai Meteorological Department http://www.tmd.go.th

[B43] Perez-Crespo M Pericuesta E Rey R Gutierrez-Adan A OC6 scrotal heat stress in mice affects viability and DNA integrity of sperm, and sex ratio of the offspring Reprod Domest Anim 2006 41 Suppl 2 104 10.1111/j.1439-0531.2006.00774_1_6.x16984480

[B44] Kozdrowski R Dubiel A The effect of season on the properties of wild boar (*Sus scrofa L*.) semen Anim Reprod Sci 2004 80 281 289 10.1016/j.anireprosci.2003.08.006 15036504

[B45] Lemma A Bekana M Schwartz HJ Hildebrandt T The effect of body condition on ovarian activity of free ranging tropical jennies (*Equus asinus*) J Vet Med A Physiol Pathol Clin Med 2006 53 1 4 16411899 10.1111/j.1439-0442.2006.00777.x

[B46] Cooper KA Harder JD Clawson DH Fredrick DL Lodge GA Peachey HC Spellmire TJ Winstel DP Serum testosterone and musth in captive male African and Asian elephants Zoo Biol 1990 9 297 306 10.1002/zoo.1430090405

[B47] Maran RR Thyroid hormones: their role in testicular steroidogenesis Arch Androl 2003 49 375 388 10.1080/713828213 12893516

[B48] Zeman M Kosutzky J Micek L Lengyel A Changes in plasma testosterone, thyroxine and triiodothyronine in relation to sperm production and remex moult in domestic ganders Reprod Nutr Dev 1990 30 549 557 10.1051/rnd:19900410 2244968

[B49] Ali H Baig M Rana MF Ali M Qasim R Khem AK Relationship of serum and seminal plasma zinc levels and serum testosterone in oligospermic and azoospermic infertile men J Coll Physicians Surg Pak 2005 15 671 673 16300697

[B50] Meeker JD Godfrey-Bailey L Hauser R Relationships between serum hormone levels and semen quality among men from an infertility clinic J Androl 2007 28 397 406 10.2164/jandrol.106.001545 17135633

[B51] Kumar N Verma RP Singh LP Varshney VP Dass RS Effect of different levels and sources of zinc supplementation on quantitative and qualitative semen attributes and serum testosterone level in crossbred cattle (Bos indicus × Bos taurus) bulls Reprod Nutr Dev 2006 46 663 675 10.1051/rnd:2006041 17169313

[B52] Strzezek J Fraser L KukliÅ„ska M DziekoÅ„ska A Lecewicz M Effects of dietary supplementation with polyunsaturated fatty acids and antioxidants on biochemical characteristics of boar semen Reprod Biol 2004 4 271 287 15592586

[B53] Mankad M Sathawara NG Doshi H Saiyed HN Kumar S Seminal plasma zinc concentration and alpha-glucosidase activity with respect to semen quality Biol Trace Elem Res 2006 110 97 106 10.1385/BTER:110:2:97 16757839

[B54] Gundogan M Elitok B Seasonal changes in reproductive parameters and seminal plasma constituents of rams in Afyon province of Turkey Dtsch Tierarztl Wochenschr 2004 111 158 161 15171601

